# A Culturally Sensitive and Theory-Based Intervention on Prevention and Management of Diabetes: A Cluster Randomized Control Trial

**DOI:** 10.3390/nu14235126

**Published:** 2022-12-02

**Authors:** Phrashiah Githinji, John A. Dawson, Duke Appiah, Chad D. Rethorst

**Affiliations:** 1 Department of Nutritional Science, Texas Tech University, 1301 Akron Ave., Lubbock, TX 79409, USA; 2 Institute of Advancing Health through Agriculture, Texas A&M AgriLife Research, 17360 Coit Rd., 17360, Dallas, TX 77843, USA; 3 Department of Economics, Applied Statistics, and International Business, New Mexico State University, 127, Las Cruces, NM 88003, USA; 4 Department of Public Health, School of Population and Public Health, Texas Tech University Health Sciences Center, 3601 4th Street, Lubbock, TX 79410, USA

**Keywords:** diabetes intervention, health belief model, culturally sensitive, diabetes prevention, health promotion, health behaviors, diet, physical activity, low and middle income and countries, community intervention

## Abstract

Type 2 diabetes is an emerging concern in Kenya. This clustered-randomized trial of peri-urban communities included a theory-based and culturally sensitive intervention to improve diabetes knowledge, health beliefs, dietary intake, physical activity, and weight status among Kenyan adults. Those in the intervention group (IG) received a culturally sensitive diabetes education intervention which applied the Health Belief Model in changing knowledge, health beliefs and behavior. Participants attended daily education sessions for 5 days, each lasting 3 h and received mobile phone messages for an additional 4 weeks. The control group (CG) received standard education on COVID-19. Data was collected at baseline, post-intervention (1 week), and follow-up assessment (5 weeks). Linear mixed effect analysis was performed to assess within and across group differences. Compared to the control, IG significantly increased diabetes knowledge (*p* < 0.001), health beliefs including perceived susceptibility (*p* = 0.05), perceived benefits (*p* = 0.04) and self-efficacy (*p* = 0.02). IG decreased consumption of oils (*p* = 0.03), refined grains (*p* = 0.01), and increased intake of fruits (*p* = 0.01). Perceived barriers, physical activity, and weight status were not significantly different between both groups. The findings demonstrate the potential of diabetes education in improving diabetes knowledge, health beliefs, and in changing dietary intake of among adults in Kenya.

## 1. Introduction

Non-communicable diseases (NCDs) are responsible for 41 million annual deaths globally [1]. Approximately 77% of NCD deaths are in low-and middle-income countries (LMICs) in South East Asia and Africa [1]. In LMICs like Kenya, NCDs are responsible for over 50% of all reported adult hospital admissions and 55% of adult mortality [2]. In these regions, the burden of morbidity and mortality due to NCDs is often overshadowed by the infectious diseases [3]. For instance, NCDs resulted in 67% of disability-adjusted life years (DALYs) and accounted for 74% of deaths globally [1,4]. However, In LMICs, NCDs receive 2% of global funds allocated from governments, the private sector, or donors in comparison to the response given to the treatments of infectious diseases such as HIV/AIDS that accounts for 3% of DALYs yet receives 30% of global funds [5,6,7,8].

About three quarters of the global burden of type 2 diabetes (T2D) occur in LMICs [9]. In Kenya, the prevalence of diabetes is 12.2% in urban areas, higher than the global prevalence of 9.3% [10]. Kenya, like other LMICs, is undergoing a nutritional and epidemiological transition as the population increases the consumption of ultra-processed, high-caloric diets and adopts a sedentary lifestyle due to social-economic development and urbanization [11]. Dietary preferences are shifting from indigenous healthier food options, rich in micro-nutrients, high fiber and diverse, including a variety of fruits, vegetables, nuts, legumes, and root tubers to western-style diets that are energy-dense and ultra-processed, high in saturated fats, added sugar and sodium [9,10]. Additionally, countries in Sub-Saharan Africa, including Kenya, now have the highest smoking and alcohol consumption rates globally [11,12].

Besides the dietary shift, changes have also occurred in physical activity patterns. For instance, there is a shift away from the high-energy expenditure activities such as farming, mining, and forestry towards the service sector and white-collar jobs, with reduced energy expenditures [13]. Similarly, economic growth, urbanization, and technological changes have also influenced and simplified how people move, with more individuals now owning cars or using train systems to conveniently get from one place to another, rather than walking [14], and this has contributed to a reduction in overall energy expenditure. In addition, unlike in the recent past, where leisure time would be spent outdoors, it is now more common that leisure time will include sedentary activities such as watching TV or smart devices [14]. Diet and physical activity transitions have influenced the demographic characteristics of those affected with T2D. For example, T2D was often associated with older age (65 years or older), but research now shows that globally, the greatest number of people with diabetes are between 40–59 years of age, with increasingly more younger people diagnosed with pre-diabetes [15].

Another reason for the increase of T2D in Kenya is a lack of knowledge about diabetes and poor health attitudes [11]. Some studies report that low awareness of risk and preventive factors for T2D significantly contribute to the increasing cases of T2D in Kenya [10,16]. People are vulnerable to misinformation, unhealthy behavior, and poor health outcomes without proper health information [17]. Furthermore, the few existing T2D programs are primarily led by the government of Kenya through the Ministry of Health (MOH) [18]. However, according to the Ministry of Health, they face several challenges that prevent successful implementation of these programs, including a lack of capacity of the health workforce in terms of numbers, equipment, and skills and poor availability and affordability of quality, safe and efficient technologies and medications for screening, diagnosis and treatment [19]. As such, many of the available documented programs on T2D education are outdated and are not supported by behavior theories that offer a framework for understanding and predicting human behavior [18,19]. Additionally, many of the existing programs lack cultural tailoring, which is proven to be important in enhancing receptivity of health programs by adapting intervention materials to fit the needs, preferences, and norms of the population more appropriately [20].

Due to the increasing prevalence of T2D enabled by the above-mentioned factors, there is a need to provide culturally sensitive diabetes education, guided by theory-based behavioral oriented methods that are effective in motivation and behavior change [21]. Consequently, this study aimed to examine the effectiveness of a theory-based and culturally sensitive educational intervention on diabetes prevention knowledge, health beliefs, dietary intake, physical activity, and weight status among adults in peri-urban communities in Nairobi, Kenya.

## 2. Materials and Methods

### 2.1. Study Design

A cluster randomized control trial (cRCT) design was used in this study. Peri-urban communities were purposively selected as study locations as they are areas where towns and the countryside meet that are nutritional zones with observable changes in diet and lifestyle and increasing growth of overweight and obesity [22]. The specific locations were peri-urban communities in Embakasi constituency, which borders the city of Nairobi and was the area that where administrative access to do the study was granted. Sample size was calculated for a cRCT with fixed number of clusters (*n* = 6) and in consideration of a power of 90%, a 5% level of significance, an expected drop-out rate of 10%, an intra-cluster correlation coefficient of 0.01, to detect a difference of 2.5 points (or a standardized effect size d of 0.8) based on a similar intervention focusing on changing diabetes knowledge and attitudes [23,24,25]. In line with these calculations, 226 participants were enrolled in the study.

Six peri-urban communities in Nairobi County were randomized so that 3 were assigned to the intervention group (IG) and 3 were assigned to the control group (this 3&3 balance was enforced by the randomization scheme). An impartial statistician with no active role in the study conducted the random assignment.

Data was collected at baseline assessment, post-intervention assessment (1 week), and at a follow-up assessment (5 weeks after baseline). Adults 18 years and older, living within peri-urban communities in Embakasi constituency were recruited through flyers distributed at the chief’s camp, religious centers, social media, and local community groups (‘Nyumba Kumi’). Those excluded were pregnant women, and those with chronic diseases, as these conditions can affect dietary intake, metabolism, and physical activity [26].

### 2.2. Diabetes Education Intervention

The diabetes education intervention was designed based on established components associated with effective health interventions for adult populations, including having clear and focused objectives and using behavioral theory to guide the intervention. The intervention focused on the desired behavior and used an interactive teaching method, such as hands-on activities and group discussions. The intervention was delivered by Community health workers (CHW) that attended a 2-day training on the delivery of the intervention. The intervention was driven by the Health Belief Model (HBM) constructs (perceived susceptibility, perceived seriousness, perceived benefits, perceived barriers, self-efficacy), and it was informed by materials from scientific sources, including the American Diabetes Association [23], the Vanderbilt Medical University Diabetes Pride Study [26] and USDA’s SNAP Education Program [27]. The intervention addressed diabetes knowledge, prevention, and management as directed by five modules: (i) understanding diabetes, (ii) preventing and managing diabetes through nutrition, (iii) physical activity, (iv) reducing tobacco and alcohol consumption, and (v) management of diabetes (Table 1). Each module was culturally tailored and designed to suit the population. For instance, the use of culturally relevant examples and use of cultural beliefs and behaviors to provide context to the content. The use of plain language, visual aids, large fonts, demonstrations were used as low health literacy strategies for teaching. Teach-back techniques were also employed to improve the understanding of diabetes concepts.

Guided by the participants’ preferred availability, those in the IG attended 5 days of concurrent face-to-face sessions, each lasting 3 h and where each of the five modules was covered. Following the completion of the face-to-face sessions, participants received three mobile-phone and WhatsApp messages each week to reinforce the materials learned, such as MyPlate, portion control and physical activity reminders. The CG received the standard education by the World Health Organization on hygiene practices related to COVID-19 [28]. The topics included hand washing and sanitization, social and physical distancing, face masks and facial coverings, cleaning protocols, assessment of symptoms and quarantine measures. To avoid contamination between the IG and CG, participants from each group met at different sites.

### 2.3. Data Collection

The lead author and 16 CHWs did all the data collection. The CHWs attended 4 h of data collection training sessions by the lead author. The training session included the purpose of the study, the role of the CHWs, the questionnaires involved and maintaining data integrity. Participant’s consent was obtained before data were collected. Participants’ anthropometrics were measured, and a set of questionnaires was administered to collect data on diabetes knowledge, health beliefs, and dietary intake and physical activity at baseline (T1), post-intervention (1 week; T2), and follow-up (5 weeks; T3).

#### 2.3.1. Knowledge Assessment

Diabetes knowledge was assessed using a questionnaire based on diabetes education objectives that reflected participants’ culture and health literacy levels. The questionnaire consisted of thirty-nine questions informed by previously validated scales [29] that sought to measure knowledge on risk factors, symptoms, the role of nutrition and physical activity, alcohol and tobacco and other self-management skills. The questionnaire underwent face and content validation by experts and was pilot tested to identify potential problem areas and deficiencies in the instrument to improve accuracy, comprehension, appropriateness, and consistency. Out of 39 possible points, participants’ knowledge was categorized as poor for scoring 13 points or less, acceptable for scoring between 14 and 26 points, and good knowledge for scoring 27 or more points.

#### 2.3.2. Health Beliefs

Health beliefs related to diabetes were assessed using a validated HBM scale from a study of the relationship between health beliefs and prevention behaviors of T2D [30]. The questionnaire had 23 items which were grouped in categories related to the constructs of the HBM. These are perceived susceptibility, perceived seriousness, perceived benefits, and perceived barriers. All questions were evaluated on a 5-point Likert scale ranging from strongly disagree (coded as 1) to strongly agree (coded as 5).

#### 2.3.3. Self-Efficacy

Self-efficacy was assessed using the validated scale from the Risk and Health Behavior Scales research [31]. The questionnaire consists of four sections with five items assessing self-efficacy related to nutrition, six items related to physical activity, five items related to alcohol and four related to smoking. The scale was based on a Likert scale of 4, where respondents are asked to rate their confidence in their ability to change their health behavior. The question responses ranged from strongly disagree (coded as 1), disagree (coded as 2), agree (coded as 3) to strongly agree (coded as 4).

#### 2.3.4. Dietary Intake

Dietary intake was assessed using a validated and culturally sensitive semi-quantitative 30-day food frequency questionnaire (FFQ) designed for adults in urban populations in Nairobi, Kenya [32]. The FFQ consists of 123 food items contributing to the total energy. The portion size was estimated by asking participants to translate their usual consumption amount based on commonly locally used utensils that were provided for demonstration. The sizes used were small (250 mL), medium (360 mL) and large (750 mL) portions. The Kenyan food composition tables were used to convert and analyze nutrient estimates for each food item [33]. The period between baseline assessment and post-intervention assessment was one week, and therefore, the dietary intake of the participants was only assessed at two-time points, at baseline and four weeks after intervention at follow-up assessment.

#### 2.3.5. Physical Activity

Physical activity was assessed using the International Physical Activity Questionnaire (IPAQ) [34]. The questionnaire consists of 27 items focusing on physical activity in household activities, activity during transportation and time spent on leisure activities. The Metabolic Equivalent of Task (METs) scores were assigned to the various activities. Physical activity was categorized as high if the participants achieved 3000 MET minutes per week with a combination of walking, moderate and vigorous activity, or three days or more of a combination of walking, moderate and vigorous activity with more than 1500 MET per week. Moderate activity consists of 600 MET per week or five or more days of any combination, including walking or moderate activity for at least 30 min per day. Participants were classified as having low activity levels if they had less than 600 MET per week and not less than 10 min of physical activity per day. Any activity less than 10 min was not considered. After that, physical activity was coded on a 3-point scale, with 1 being high, 2 moderate physical activity and 3-low physical activity.

#### 2.3.6. Anthropometric Measures

The body weight of each participant was measured using a properly calibrated digital scale, and their information was recorded in kilograms in their individually labeled questionnaires. Weight was later converted to pounds. Height was measured in centimeters using a wall-mounted stadiometer and later converted to inches.

### 2.4. Data Analysis

Data were analyzed using SPSS system (Version 28, SPSS Inc, Chicago, IL, USA). Descriptive statistics were used to describe differences in demographic characteristics, knowledge, health beliefs, household food insecurity, physical activity, and dietary intake among groups. One-way Analysis of Variance (ANOVA) and chi-square (*χ^2^*) tests were used to assess for equivalency between the demographic variables (age, income, marital status, education level, BMI) between the IG and CG. Linear mixed effect models with random effects for clusters and subjects were fit to assess within and between group differences in the outcomes from baseline to post-intervention and from to follow-up assessment. The change scores in diabetes knowledge, dietary intake, health beliefs, physical activity and weight status was compared between the IG (Δ_IG_: change score in the IG) and CG (Δ_CG_: change score in the CG). The linear mixed effect analysis adjusted for age and BMI. An alpha level of 0.05 was used as the threshold for statistical significance.

## 3. Results

**Figure 1:**
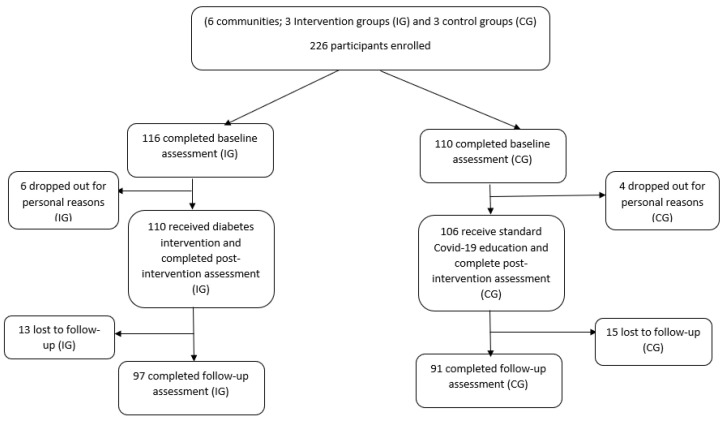
Flow diagram of recruitment and study participation.

A total of 226 adults completed the baseline assessment (116 adults in the IG; 110 in the CG). Ten participants (6 in the IG and 4 in the CG) dropped out of the study for personal reasons. A total of 216 adults attended the study and completed the post-test assessment (T2). An additional 28 adults (13 in the IG; 15 in the CG) dropped out from the study before the follow-up assessment. A total of 188 adults (83.2%) completed the study in its entirety (Figure 1).

A summary of the characteristics of the participants can be found in Table 1. Most participants were females (71.7%), with a mean age of 37.5 years with majority (54.5%) having overweight or obesity (54.5%). Statistical comparisons showed significant differences in age and BMI between the two groups (*p* =0.02; *p* < 0.001) that were adjusted for in analysis. No other differences between the groups were significant (Table 1).

### 3.1. Effect of Intervention on Diabeteknowledge, Health beliefs, Physical Activity, Dietary Intake and Weight Status

#### 3.1.1. Diabetes Knowledge

The mean diabetes knowledge score of participants in the IG at baseline was 1.43. After the intervention, this significantly increased to 2.80 at post-intervention and to 2.87 at follow-up. Compared to the CG, this increase in knowledge in the IG was significant over time from T1 to T3 (*p <* 0.001) (Table 2) and across the other assessment time points (Tables S1 and S2).

#### 3.1.2. Health Beliefs

##### Perceived Susceptibility

On a scale of 5, the perceived susceptibility to T2D in the IG significantly increased from 2.37 to 3.03 to 3.22 points (Table 2). Comparing the IG and the CG over the three assessment periods, there was a significant increase in the perceived susceptibility of participants in the IG versus the CG from T1 to T3 (*p =* 0.05; Table 2) and across the other assessment time points (Tables S1 and S2).

##### Perceived Seriousness

There was no significant change in the perceived seriousness of diabetes in both groups. At baseline, the mean score for the IG was 2.91 points, which changed to 2.53 at the post-test and 2.51 at the follow-up assessment. When comparing the two groups, the change in perceived seriousness was not significant for participants in the IG versus the CG at any of the three time points (Table 2, Tables S1 and S2).

##### Perceived Benefits

Perceived benefits of adopting healthier behaviors among the intervention group significantly increased in this study. For example, at baseline, IG participants scored an average of 3.63, 4.30 points at post intervention and 3.64 points at follow up assessment. This was a significant increase in perceived benefits of participants compared to the CG from T1 to T3 (*p* = 0.04; Table 2) and across the other assessment time points (Tables S1 and S2).

##### Perceived Barriers

The participant’s perceived barriers to achieving a healthy lifestyle did not significantly change within and across groups (Table 2). The mean score for the IG was 2.58, 2.54, and 2.45 at the three respective time points, and this was not statistically different at any time. (Table 2, Tables S1 and S2).

##### Self-Efficacy

Self-efficacy related to nutrition and healthy eating significantly increased in the IG over the three assessment points from 3.12 to 3.82 to 3.86 points, respectively. When comparing the two groups over the three assessment periods, there was a significant increase in the self-efficacy (nutrition) of participants in the IG versus the CG (Table 2, Tables S1 and S2). The mean self-efficacy at baseline for those in the IG who consumed alcohol was 3.08 points at baseline, 3.49 at post-test and 3.48 at follow-up assessment. Self-efficacy for the CG was 3.04 at baseline, 2.99 at post-test and 2.86 at follow-up assessment. In comparison, there was a significant difference in self-efficacy relating to alcohol use by participants in the IG versus the CG from T1 to T3 (*p =* 0.02) and across the other assessment (Tables S1 and S2). Self-efficacy related to smoking and physical activity did not significantly increase for participants within and across both groups from T1 to T2 to T3 (Table 2).

#### 3.1.3. Dietary Intake

As shown in Table 3, the intake of refined grains in the IG significantly decreased from a mean intake of 6.03 cups at baseline assessment to 5.43 cups at follow-up assessment *p =* 0.004). The CG refined grain intake was 6.01 cups at baseline and 6.13 at follow-up (*p =* 0.363). The IG had a significantly reduced intake of refined grains versus the CG (*p =* 0.009). The mean daily intake of oils in the IG significantly decreased from 42.53 g at baseline to 38.7 g at follow-up assessment (*p <* 0.001). While the CG intake of oils did not significantly change from 43.54 g at baseline and 44.72 g at follow-up assessment. Overall, the IG had a significantly reduced intake of oils versus the CG (*p =* 0.030). Additionally, mean daily fruit intake significantly increased for participants in the IG from 3.68 to 4.66 cups (*p =* 0.002) but did not significantly increase for the CG (*p =* 0.663). The dairy intake was 5.14 at baseline, and 4.41 cups at follow-up assessment (*p =* 0.03), and this was different compared to CG over time (*p =* 0.05) (Table 3).

#### 3.1.4. Physical Activity and Weight Status

Physical activity measured in METs was categorized on a 3-point scale, with 1 being high, 2 moderate and 3-low physical activity. The mean scores on physical activity among participants in the IG were 1.92 at baseline, 1.96 at post-test and 1.89 at follow-up assessment (*p =* 0.96). The mean score for the CG was 1.90 at baseline, 1.94 at post-test, and 1.91 at follow-up assessment (*p =* 0.99). The mean weight for the IG was 164.16 at baseline, 162.26 at post-test and 166.96 pounds at follow-up assessment. The mean weight for the CG was 174.34, 175.45, and 179.45 pounds within the three respective time points. There were no significant differences in changes in weight status within and across groups from baseline to post-intervention and follow-up assessment (*p =* 0.96).

## 4. Discussion

Findings from this study showed that a theory-based intervention was effective in increasing diabetes knowledge and improving health beliefs (perceived susceptibility to diabetes, perceived benefits, and self-efficacy) in adopting a healthier lifestyle. The intervention also improved dietary intake with reduced intake of refined grains and oils and increased intake of fruits. However, the intervention did not result in a significant improvement in the other food groups, perceived seriousness, perceived barriers, physical activity and weight status.

The findings of this study showed an improvement in knowledge of diabetes risk factors, symptoms, role of nutrition and physical activity, alcohol and tobacco and other self-management skills. These findings are similar to other studies conducted in LMICs, including by Muchiri and colleagues, where participants in the treatment group who received a diabetes education intervention had higher knowledge scores than the control group [23]. Similarly, Chawla and colleagues found that participants that received education showed a significant increase in knowledge from baseline to endpoint compared to the control group [35]. Diabetes education interventions can lead to an increase in knowledge, as demonstrated by a systematic review that looked at over 19 heterogeneous trials and found that education interventions led to a significant increase in knowledge of T2D [35].

Perceived susceptibility to diabetes, perceived benefits and self-efficacy improved in this study. However, perceived seriousness and barriers did not improve. Like these findings, several studies have found that while perceived susceptibility, benefits, and self–efficacy improve, there may be an inverse relationship with perceived seriousness. For instance, in a similar study where participants received an education intervention on calcium intake, the perceived seriousness of osteoporosis declined with increased knowledge, improved benefits and self-efficacy [36]. Likewise, Suratman and colleagues, in an educational intervention to improve perceptions for reducing exposure to pesticides in farmers, found a decrease in perceived seriousness with improved perceived susceptibility [37]. It is possible that as participants increase their knowledge and perceived benefits of adopting healthier behaviors and improve their self-efficacy, these improvements contribute to a decrease in the perceived seriousness of disease as participants feel empowered by the knowledge and skills to change their behavior to a healthier lifestyle.

This study looked at barriers at the inter-personal level in the socio-ecological context, such as taste, time management, cost of food, social influences from family and friends, motivation, and stress [38,39]. These perceived barriers did not significantly improve. A possible reason for the lack of change is that the study was conducted during the COVID-19 pandemic and effects of the lockdowns could have exacerbated health barriers. Furthermore, barriers addressed were at the individual level, and yet there may be others at the macro level, such as lack of employment opportunities, gender inequalities, inhibitive policies, poverty, and insecurity [40]. Therefore, future studies can incorporate a broader scope while addressing perceived barriers to diabetes health.

The intake of whole grains was low at 2.4 cups compared to the recommended 2.5–4.5 cup equivalents per day. Refined grain intake was high, with average intake of 6.03 cups compared to the recommended intake of 3.5–4.5 cup eq/day. These findings are consistent with other studies investigating dietary patterns that have found that the average diet in Nairobi, Kenya is heavily reliant on carbohydrates as a source of energy intake with primary staples being maize products, wheat, rice and cooking bananas [41]. A time series study assessing staple food consumption patterns in households in Nairobi, and its environs, found that there was a significant increase in consumption of milled and refined grains overtime at the expense of whole grains [41]. This could explain the high intake of refined carbohydrates versus whole grains in this study. The intake of refined grains significantly reduced after the diabetes intervention, and this was ultimately a positive effect as there’s higher risks of obesity and associated chronic diseases, with increased intake of refined grains [42]. However, while refined grains reduced, the intake of starchy vegetables increased. This may signify a dietary compensation mechanism that has been observed in some studies, and that needs further investigation, where in response to adjustments in energy intake, individuals compensate by increasing intake of certain foods to maintain satiety and regardless of portion control there’s constant energy intake [43]. The high mean intake of dark green vegetables (4.95 vs. 2.0–2.5 cups/week), red and orange vegetables (6.15 vs. 6.0 cups/week), and fruits (3.68 vs. 2.0 cups/day) at baseline could explain the lack of significant change in intake after the intervention [44]. Nyanchoka and colleagues had similar findings in a study in Kenya where 78% of the respondents met fruits, vegetables and beans/peas/lentils recommendations [45].Regarding the lack of change in physical activity, participants in this study already had a high physical activity of 3000 MET minutes per week with a combination of walking and moderate and vigorous activity at baseline assessment. It is possible that this contributed to the lack of observable change in the amount of physical activity at post-test and follow-up assessment. Weight status did not change significantly after the intervention. Notedly, there was a significant portion of the sample in the underweight and normal weight categories, and this could have statistically affected the ability to observe significant change in weight. Additionally, weight loss is extremely challenging due to interactions between our biology, behavior, and obesogenic environments [46]. Change in body weight also requires consistent behavior change and long-term observation, which could explain the lack of significant change in this study [47]. It is recommended that future interventions that hope to observe a significant change in weight status have a longer post-intervention period, preferably longer than the four weeks applied in this study.

### Implications and Recommendations

The findings of this study have several implications and recommendations for policy and future research. The increase in diabetes knowledge, perceived susceptibility, benefits, and self-efficacy after the intervention, amplifies the importance of educating people to prevent and manage T2D. Education interventions should target improving awareness of T2D and increasing perceived threats to diabetes, and the benefits of adopting healthier behavior while addressing barriers and building self-efficacy.

The CHWs were instrumental in implementing the intervention, since they are well known to the community, they can be tapped into to educate the community on T2D as a sustainable measure and can facilitate the scaling up of existing or future T2D programs. Lastly, the cultural and theory-based aspects of the intervention were a crucial part of ensuring that there was increased knowledge, perceived susceptibility, perceived benefits, self-efficacy and improving dietary intake. These have important implications for the designing of future community-based health interventions focusing on T2D prevention and management. It is recommended that similar health education interventions be culturally tailored, theory-driven and should also apply low health literacy strategies. They should also extend their duration to measure sustained behavioral changes.

This study had some limitations. For instance, some data were self-reported and may be subject to bias resulting from recall or social desirability, especially in reporting dietary intake data and health beliefs. Additionally, while the diabetes knowledge questionnaire was pilot tested and underwent face and content validation by experts, it may benefit from further confirmatory analyses to establish reliability before use in other settings. Again, the study was not powered to detect non-primary outcomes, which may have affected our ability to observe changes in physical activity and body weight. Lastly, the short study period may have limited the ability to detect body weight changes and hindered the evaluation of the long-term effects of the intervention effects.

## 5. Conclusions

The findings of this study demonstrate that the culturally sensitive and theory-based diabetes intervention was effective in increasing diabetes knowledge and improving health beliefs, including perceived seriousness, perceived benefits, and self-efficacy. It also effectively improved the dietary intake of refined grains, oils, fruits, and dairy among adults in peri-urban communities in Nairobi, Kenya.

## Figures and Tables

**Table 1:** Demographic characteristics of participants (*n* = 226).

	Total Participants	Intervention Group	Control Group	*p*-Value
Mean ± SD				
Age (years)	37.5 ± 12.7	39.4 ± 12.6	35.5 ± 12.7	0.02
Weight (pounds)	169.9 ± 38.8	164.2 ± 31.1	175.9 ± 44.8	0.02
Height (inches)	65.0 ± 6.4	63.7 ± 8.5	66.0 ± 3.4	0.01
Body Mass Index				
Underweight	4 (1.8)	3 (2.6)	1 (0.9)	0.001
Normal weight	84 (37.2)	40 (34.8)	44 (40.0)	
Overweight	51 (22.6)	22 (19.1)	29 (26.4)	
Obese	72 (31.9)	36 (31.3)	36 (32.7)	
Gender				
Male	64 (28.3)	38 (32.8)	26 (23.6)	0.08
Female	158 (69.9)	74 (63.8)	84 (76.4)	
Education				
Primary School	9 (4.0)	5 (4.3)	4 (3.6)	0.06
Secondary School	77 (34.1)	28 (24.1)	49 (44.5)	
College (2-year degree)	77 (34.1)	45 (38.8)	32 (29.1)	
University Degree (4-year degree)	50 (22.1)	29 (25.0)	21 (19.1)	
Post-graduate Degree	12 (5.3)	8 (6.9)	4 (3.6)	
Occupation				
Government employed	87 (38.5)	49 (42.2)	38 (34.5)	0.81
NGO employed	27 (11.9)	14 (12.1)	13 (11.8)	
Self employed	43 (19.0)	21 (18.1)	22 (20.0)	
Homemaker	9 (4.0)	3 (2.6)	6 (5.5)	
Retired	7 (3.1)	4 (3.4)	3 (2.7)	
Not employed	50 (22.1)	23 (19.8)	27 (24.5)	
Unable to work	2 (0.9)	1 (0.9)	1 (0.9)	
Household Income				
100 USD or less	49 (21.7)	24 (20.7)	25 (22.7)	0.97
100–299 USD	75 (33.2)	38 (32.8)	37 (33.6)	
300–499 USD	41 (18.1)	21 (18.1)	20 (18.2)	
500–999 USD	35 (15.5)	19 (16.4)	16 (14.5)	
1000 USD and above	9 (4.0)	5 (4.3)	4 (3.6)	
Diabetes Diagnosis				
Yes	20 (8.8)	9 (45.0)	11 (55.0)	0.71
No	206 (91.2)	107 (51.9)	99 (48.1)	
Family history of diabetes				
Yes	75 (33.2)	36 (48.0)	39 (52.0)	0.67
No	151 (66.8%)	71 (47.0)	80 (53.0)	
Type of family member with diabetes				
dia	32 (42.6%)	14 (43.8)	18 (56.2	0.63
Extended	28 (37.33%)	17 (60.7)	11 (39.30)	
Both	15 (20.0%)	6 (40)	9 (60)	

**Table 2:** Changes in diabetes knowledge and health beliefs from baseline (T1) to post-intervention (T2) and follow-up (T3) assessments.

Variables	Baseline Assessment (T1)		Post-Intervention Assessment (T2) (1 Week after Baseline)		Follow-Up Assessment (T3) (5 Weeks after Baseline)		Δ_T3-T1_ (m ± sd)		*p*-Value ^†^ between Groups over Time
	Mean Change ± SD		Mean Change ± SD		Mean Change ± SD				
	IG	CG	IG	CG	IG	CG	IG	CG	
Diabetes Knowledge	1.43 ± 0.68	1.36 ± 0.61	2.80 ± 0.55	1.31 ± 0.59	2.87 ± 0.43	1.40 ± 0.69	1.44 ± 0.56	0.04 ± 0.65	0.001
Perceived Susceptibility	2.37 ± 1.06	2.21 ± 1.02	3.03 ± 0.53	2.27 ± 0.95	3.22 ± 1.11	2.34 ± 0.90	0.85 ± 1.09	0.13 ± 0.96	0.05
Perceived Seriousness	2.91 ± 0.83	3.13 ± 0.72	2.53 ± 0.39	3.03 ± 0.79	2.51 ± 0.97	2.99 ± 0.78	−0.4 ± 0.90	−0.14 ± 0.75	0.06
Perceived Benefits	3.63 ± 0.94	3.48 ± 0.95	4.30 ± 0.91	3.41 ± 0.89	3.64 ± 0.89	3.43 ± 0.85	0.01 ± 0.92	−0.05 ± 0.90	0.04
Perceived Barriers	2.58 ± 0.92	2.67 ± 0.92	2.54 ± 0.32	2.64 ± 0.81	2.45 ± 0.94	2.68 ± 0.75	−0.13 ± 0.93	0.01 ± 0.84	0.09
Self-efficacy-Nutrition	3.12 ± 0.74	3.09 ± 0.76	3.82 ± 0.62	3.08 ± 0.68	3.86 ± 0.72	3.06 ± 0.68	0.74 ± 0.73	−0.03 ± 0.72	0.02
Self-efficacy-Physical activity	2.98 ± 0.82	2.92 ± 0.83	3.03 ± 0.82	2.89 ± 0.79	3.05 ± 0.81	2.87 ± 0.77	0.07 ± 0.82	−0.05 ± 0.80	0.98
Self-efficacy-Alcohol (*n* = 76)	3.08 ± 0.81	3.04 ± 0.87	3.49 ± 0.29	2.99 ± 0.82	3.48 ± 0.81	2.86 ± 0.83	0.40 ± 0.81	−0.18 ± 0.85	0.02
Self-efficacy-Smoking (*n* = 30)	2.71 ± 1.02	2.33 ± 0.88	2.85 ± 0.32	2.89 ± 1.01	2.71 ± 1.02	2.89 ± 1.02	0.00 ± 1.02	0.56 ± 0.95	0.12

Note: Δ_T3-T1_: change score from T1 to T3 assessment, calculated by subtracting score at T1 from score at T3; IG: intervention group; CG: control group. ^†^ Adjusted for cluster differences in age, and BMI.

**Table 3:** Changes in food groups consumption from baseline (T1) to follow-up assessment (T3).

Food Groups	Baseline Assessment Mean (SD)		Follow-Up Assessment Mean (SD)		Δ_T3-T1_ (m ± sd)		*p*-Value between Groups Over Time
	IG	CG	IG	CG	IG	CG	
Whole Grain ^††^	2.40 (2.26)	2.28 (2.23)	2.09 (2.51)	2.30 (2.31)	−0.31 ± 2.385	0.02 ± 2.27	0.77
Refined Grains ^††^	6.03 (1.89)	6.01 (1.92)	5.43 (1.29)	6.13 (1.03)	−0.6 ± 1.59	0.12 ± 1.48	0.01
Meat/poultry/eggs ^φ^	1.48 (1.68)	1.52 (1.68)	2.38 (1.39)	1.72 (1.36)	0.9 ± 1.54	0.20 ± 1.52	0.34
Fish/seafood ^φ^	3.36 (1.67)	3.01 (1.67)	3.22 (1.29)	3.38 (1.23)	−0.14 ± 1.48	0.37 ± 1.45	0.69
Dark green vegetable ^Φ^	4.93 (2.21)	4.95 (2.22)	4.84 (1.49)	4.56 (1.81)	−0.09 ± 1.85	−0.39 ± 2.02	0.17
Red & orange vegetables ^Φ^	6.15 (1.87)	6.14 (1.89)	6.23 (0.91)	6.22 (0.83)	0.08 ± 1.39	0.08 ± 1.36	0.56
Other vegetables ^Φ^	3.98 (2.60)	3.99 (2.62)	3.86 (2.17)	3.87 (1.99)	−0.12 ± 2.39	−0.12 ± 2.31	0.51
Oils ^¥^	42.53 (1.42)	43.54 (1.02)	38.7 (0.83)	44.72 (0.86)	−3.83 ± 1.13	1.18 ± 0.94	0.03
Starchy vegetables ^Φ^	2.21 (0.31)	2.28 (0.31)	3.62 (0.57)	2.26 (3.09)	1.41 ± 0.44	−0.02 ± 1.70	0.001
Beans/peas/lentils ^Φ^	2.19 (2.64)	2.17 (2.65)	2.47 (2.17)	2.37 (1.95)	0.28 ± 2.41	0.20 ± 2.30	0.29
Fruits ^††^	3.68 (2.88)	3.66 (2.93)	4.66 (1.88)	3.81 (2.00)	0.98 ± 2.38	0.15 ± 2.47	0.01
Dairy ^††^	5.14 (3.02)	5.02 (3.08)	4.41 (2.00)	4.92 (2.20)	−0.73 ± 2.50	−0.10 ± 2.64	0.05
Nuts/seeds/soy ^φ^	0.75 (1.20)	0.82 (1.33)	0.78 (0.89)	0.75 (0.85)	0.03 ± 1.05	−0.07 ± 1.09	0.87

Note: Δ_T3-T1_: change score from T1 to T3 assessment, calculated by subtracting score at T1 from score at T3; IG: intervention group; CG: control group. ^φ^ demotes dietary intake reported in ounce equivalents per day. ^¥^ demotes dietary intake reported in grams per day. ^Φ^ denotes dietary intake reported in cup equivalents per week. ^††^ denotes dietary intake reported in cup equivalents per day. Food group intake based on participant’s mean caloric intake of 2200–2600.

## Supplementary Materials

The following supporting information can be downloaded at: https://www.mdpi.com/article/10.3390/nu14235126/s1, Table S1: Changes in diabetes knowledge and health beliefs from baseline (T1) to post-intervention assessment (T2); Table S2: Changes in diabetes knowledge and health beliefs from post-intervention (T2) to follow-up assessment (T3).
