# Supplementary material for: A Culturally Sensitive and Theory-Based Intervention on Prevention and Management of Diabetes: A Cluster Randomized Control Trial
Source: Nutrients. 2022 Dec 2;14(23):5126. doi: 10.3390/nu14235126 (PMC9737926; doi:10.3390/nu14235126)
Supplement: Supplementary file 1 [file nutrients-14-05126-s001.zip › nutrients-2031186-supplementary.pdf]

Article

A Culturally Sensitive and Theory-Based Intervention on Prevention and Management of Diabetes: A Cluster Randomized Control Trial

Phrashiah Githinji[1,2*], John Dawson[1,3], Duke Appiah[4] and Chad Rethorst[2]

[1] Department of Nutritional Science, Texas Tech University, 1301 Akron Ave., Lubbock, TX 79409, USA
[2] Institute of Advancing Health through Agriculture, Texas A&M AgriLife Research, 17360 Coit Rd., 17360, Dallas, TX 77843, USA
[3] Department of Economics, Applied Statistics, and International Business, New Mexico State University, 127, Las Cruces, NM 88003, USA
[4] Department of Public Health, School of Population and Public Health, Texas Tech University Health Sciences Center, 3601 4th Street, Lubbock, TX 79410, USA
* Correspondence: phrashiah.githinji@ag.tamu.edu

Citation: Githinji, P.; Dawson, J. A; Appiah, D.; Rethorst, C. D. Culturally Sensitive and Theory-Based Intervention on Prevention and Management of Diabetes: A Cluster Randomized Control Trial. *Nutrients* **2022**, *14*, 5126. https://doi.org/10.3390/nu14235126

Academic Editor: Carlos Vasconcelos

Received: 29 October 2022
Accepted: 30 November 2022
Published: 2 December 2022

Publisher's Note: MDPI stays neutral with regard to jurisdictional claims in published maps and institutional affiliations.

Copyright: © 2022 by the authors. Licensee MDPI, Basel, Switzerland. This article is an open access article distributed under the terms and conditions of the Creative Commons Attribution (CC BY) license (https://creativecommons.org/licenses/by/4.0/).

Supplementary Materials

Table S1. Changes in diabetes knowledge and health beliefs from baseline (T1) to post-intervention assessment (T2)

**Variable**	**Baseline assessment (T1)**		**Postintervention assessment (T2) (1 week after baseline)**		**Δ_{T2-T1} (m ± sd)**		**P-value† Between Groups over time**
	**Mean change ± SD**		**Mean change ± SD**				
	**IG**	**CG**	**IG**	**CG**	**IG**	**CG**	
Diabetes Knowledge	1.43 ±0.68	1.36 ±0.61	2.80 ±0.55	1.31 ±0.59	1.37± 0.55	-0.05 ± 0.60	**0.001**
Perceived Susceptibility	2.37 ± 1.06	2.21 ± 1.02	3.03 ± 0.53	2.27 ± 0.95	0.66± 0.53	-0.06 ± 0.99	**0.01**
Perceived Seriousness	2.91 ± 0.83	3.13 ± 0.72	2.53 ± 0.39	3.03 ± 0.79	--0.38±0.61	-0.10 ± 0.76	0.06
Perceived Benefits	3.63 ± 0.94	3.48 ± 0.95	4.30 ± 0.91	3.41 ± 0.89	0.67 ± 0.93	-0.07 ± 0.92	**0.01**
Perceived Barriers	2.58 ± 0.92	2.67 ± 0.92	2.54 ± 0.32	2.64 ± 0.81	-0.04 ± 0.62	-0.03 ± 0.87	0.88
Self-efficacy – Nutrition	3.12 ± 0.74	3.09 ± 0.76	3.82 ± 0.62	3.08 ± 0.68	0.70 ± 0.68	-0.01 ± 0.72	**0.04**
Self-efficacy - Physical activity	2.98 ± 0.82	2.92 ± 0.83	3.03 ± 0.82	2.89 ± 0.79	0.05 ± 0.82	-0.03 ± 0.81	0.63
Self-efficacy - Alcohol (n=76)	3.08 ± 0.81	3.04 ± 0.87	3.49 ± 0.29	2.99 ± 0.82	0.41 ± 0.55	-0.05 ± 0.85	**0.05**
Self-efficacy - Smoking (n=30)	2.71 ± 1.02	2.33 ± 0.88	2.85 ± 0.32	2.89 ± 1.01	0.14 ± 0.67	0.56 ± 0.99	0.37

Note: Δ_{T2-T1}: change score from T1 to T2 assessment, calculated by subtracting score at T1 from score at T2; IG: intervention group; CG: control group.

† Adjusted for cluster differences in age, and BMI

Table S2. Changes in diabetes knowledge and health beliefs from post-intervention (T2) to follow-up assessment (T3)

Variables	Post-intervention assessment (T2)		Follow-up assessment (T3)		Δ_{T3-T2} (m ± sd)		P-value† Between Groups over time
	Mean change ± SD		Mean change ± SD				
	IG	CG	IG	CG	IG	CG	
Diabetes Knowledge	2.80 ±0.55	1.31 ±0.59	2.87 ±0.43	1.40 ±0.69	0.07 ± 0.49	0.09 ± 0.64	**0.001**
Perceived Susceptibility	3.03 ± 0.53	2.27 ± 0.95	3.22 ± 1.11	2.34 ± 0.90	0.19 ± 0.82	0.07 ± 0.93	**0.01**
Perceived Seriousness	2.53 ± 0.39	3.03 ± 0.79	2.51 ± 0.97	2.99 ± 0.78	-0.02 ± 0.68	-0.04 ± 0.79	0.06
Perceived Benefits	4.30 ± 0.91	3.41 ± 0.89	3.64 ± 0.89	3.43 ± 0.85	-0.66 ± 0.90	0.02 ± 0.87	**0.01**
Perceived Barriers	2.54 ± 0.32	2.64 ± 0.81	2.45 ± 0.94	2.68 ± 0.75	-0.09 ± 0.63	0.04 ± 0.78	0.85
Self-efficacy – Nutrition	3.82 ± 0.62	3.08 ± 0.68	3.86 ± 0.72	3.06 ± 0.68	0.04 ± 0.67	-0.02 ± 0.68	**0.01**
Self-efficacy - Physical activity	3.03 ± 0.82	2.89 ± 0.79	3.05 ± 0.81	2.87 ± 0.77	0.02±0.82	-0.02 ± 0.78	0.72
Self-efficacy - Alcohol (n=76)	3.49 ± 0.29	2.99 ± 0.82	3.48 ± 0.81	2.86 ± 0.83	-0.01±0.55	-0.13 ± 0.83	**0.03**
Self-efficacy - Smoking (n=30)	2.85 ± 0.32	2.89 ± 1.01	2.71 ± 1.02	2.89 ± 1.02	-0.14±0.67	0.00 ± 1.02	0.14

Note: Δ_{T3-T2}: change score from T2 to T3 assessment, calculated by subtracting score at T2 from score at T3; IG: intervention group; CG: control group.

† Adjusted for cluster differences in age, and BMI
